# The relationship of high sensitivity C-reactive protein to percent body fat mass, body mass index, waist-to-hip ratio, and waist circumference in a Taiwanese population

**DOI:** 10.1186/1471-2458-10-579

**Published:** 2010-09-28

**Authors:** Cheng-Chieh Lin, Sharon LR Kardia, Chia-Ing Li, Chiu-Shong Liu, Ming-May Lai, Wen-Yuan Lin, Pei-Chia Chang, Yih-Dar Lee, Ching-Chu Chen, Chih-Hsueh Lin, Chuan-Wei Yang, Chih-Yi Hsiao, Walter Chen, Tsai-Chung Li

**Affiliations:** 1 Department of Family Medicine, China Medical University & Hospital, Taichung, Taiwan; 2 Medical Research, China Medical University & Hospital, Taichung, Taiwan; 3 Department of Family Medicine, College of Medicine, China Medical University & Hospital, Taichung, Taiwan; 4 Institute of Health Care Administration, College of Public Health, China Medical University & Hospital, Taichung, Taiwan; 5 Department of Healthcare Administration, College of Health Science, Asia University, Taichung, Taiwan; 6 Department of Epidemiology, University of Michigan, Ann Arbor, Michigan, USA; 7 Administration Center, China Medical University & Hospital, Taichung, Taiwan; 8 Department of Psychiatry, Medical College, National Cheng-Kung University, Tainan, Taiwan; 9 Bristol-Myers Squibb (Taiwan) Ltd, Global Development & Medical Affairs, Taipei, Taiwan; 10 Division of Endocrinology and Metabolism, Department of Medicine, China Medical University & Hospital, Taichung, Taiwan; 11 Department of Medicine, China Medical University & Hospital, Taichung, Taiwan; 12 Graduate Institute of Biostatistics & Chinese Medicine Science, China Medical University & Hospital, Taichung, Taiwan; 13 Biostatistics Center, China Medical University & Hospital, Taichung, Taiwan

## Abstract

**Background:**

High-sensitivity C-reactive protein (hs-CRP) is an easily measured inflammatory biomarker. This study compared the association of percent body fat mass (%FM), body mass index (BMI), waist circumference (WC), and waist-to-hip ratio (WHR) with hs-CRP in a Taiwanese population.

**Methods:**

A total of 1669 subjects aged 40-88 years were recruited in 2004 in a metropolitan city in Taiwan. The relationships between obesity indicators and a high level of hs-CRP were examined using multivariate logistic regression analysis. The upper quartile of the hs-CRP distributions was defined as the high category group. The areas under the curve (AUCs) of the receiver operating characteristic curves were calculated for all obesity indicators to compare their relative ability to correctly classify subjects with a high level of hs-CRP.

**Results:**

After multivariate adjustment, the odds ratio for %FM was the only significant indicator that was associated with a high level of hs-CRP in men (1.55, 95% CI: 1.07-2.25). All indicators were associated with a high level of hs-CRP in women. In men, the AUCs for %FM were significantly higher than those for BMI, WHR, and WC, when demographic and lifestyle behaviors were considered (p < 0.001 for all comparisons), but they were not significantly different in females.

**Conclusions:**

Our study demonstrates that %FM is the only obesity indicator that is strongly associated with a high level of hs-CRP after adjusting for sociodemographic factors, lifestyle behaviors and components of metabolic syndrome in both genders in a Taiwanese population aged forty years and over. In men, %FM had the greatest ability to classify subjects with a high level of hs-CRP when only demographic and lifestyle behaviors were considered. Our study finding has important implications for the screening of obesity in community settings.

## Background

Obesity is a major public health problem in the world, affecting people in both developed and developing countries [1-4]. According to reports, there are about 250 million adults suffering from obesity [5,6]. A previous survey has shown that the age-adjusted prevalence of obesity is rapidly increasing in Taiwan, from 10.5% to 15.9% for men from 1993-1996 to 2000-2001 [7]. Of most importance, obesity is associated with numerous chronic health conditions or diseases, such as diabetes, hypertension, and cardiovascular disease [8,9].

High-sensitivity C-reactive protein (hs-CRP), the main acute phase protein in humans, is a sensitive marker for systemic inflammation. Previous cross-sectional studies have shown that elevated hs-CRP levels correlate significantly with features of metabolic abnormality, including adiposity, hyperinsulinemia, insulin resistance, hypertriglyceridemia, and low high-density lipoprotein cholesterol (HDL-C) [10-13]. Although the physiological mechanisms linking elevated hs-CRP to these disorders are not known, it is possible that the association is partly mediated by adipose tissue, a main source of inflammatory cytokines. Most epidemiologic studies identifying strong associations between hs-CRP and obesity indicators predominantly use anthropometric indexes [10-13]. Only a few studies have used measures of body fat [14-16].

Body mass index (BMI) is an indicator of heaviness rather than fatness, and cannot distinguish body fat from fat-free mass. Waist-to-hip ratio (WHR) is a measure of regional fat distribution, whereas waist circumference (WC) is a measure of central obesity. Bio-electrical impedance analysis (BIA) produces a close estimate of fat-free mass throughout a wide range of body composition [17]. BIA is a non-invasive measurement of body composition and is especially useful in large epidemiologic studies. It also possesses many advantages over other methods, in that it is inexpensive, simple, fast, safe, portable, easy-to-perform, and requires minimal operator training [18,19]. The current study was designed to explore the values of obesity indicators of percent body fat mass (%FM), as determined by BIA for the total body, compared to BMI, WC, and WHR, in terms of their independent relation to hs-CRP under two conditions: one considering metabolic syndrome (MetS)'s components, except obesity, and the other not considering these MetS components.

## Methods

### Participants

This was a community-based cross-sectional study based on data from the Taichung Community Health Study. The detailed methodology has been described elsewhere [20-25]. A total of 2,359 residents of Taichung City, Taiwan, aged 40 and over, participated in the study in October 2004. Hs-CRP levels were measured only for the first 1669 consecutive subjects. This study was approved by the Human Research Committee of China Medical University Hospital. Written informed consent was obtained from each participant.

### Anthropometric measurements and laboratory examinations

Anthropometric measurements and blood samples were obtained from the complete physical examination. Weight and height were measured on an auto-anthropometer (super-view, HW-666), with the subjects shoeless and wearing light clothing. BMI was derived from the formula, weight (kg) ÷ (height)^2 ^(m^2^). With the participant standing, WC was measured midway between the superior iliac crest and the costal margin, and hip circumference at its maximum protrusion point of the buttocks around the pelvis, and then the WHR was calculated as a measure of regional fat distribution. %FM was assessed by a body composition analyzer (Tanita BC-418, Arlington Heights, Illinois, USA). The measurements were performed with the subjects stepping onto the measuring platform without shoes and after wiping the soles of their feet. The amount of body fat was expressed as a percentage of total weight. Previous studies have confirmed the validity of BIA in estimating body composition compared to dual energy X-ray absorptiometry (DEXA) [19,26]. The means values for BIA and DEXA were very close [26]. BIA was a good predictor of DEXA-derived fat-free mass (r = 0.85-0.88) and was superior to BMI in measuring body fat [19]. The principle of BIA is that fat tissue exhibits greater resistance to the flow of electrical current than fat-free tissue because of differences in water content.

Blood pressure was measured by an electronic device (COLIN, VP-1000, Japan) three times after the subjects had rested for 20 minutes. The lowest systolic and diastolic blood pressure was recorded. Blood was drawn from an antecubital vein in the morning after a 12-hour overnight fasting and was sent for analysis within four hours of blood collection. Hs-CRP levels were measured by nephelometry, a latex particle-enhanced immunoassay (TBA-200FR, Tokyo, Japan). The interassay and intra-assay CVs were <2.0% and <1.9%, respectively. The lower detection limit of the assay was 0.1 mg/L. Biochemical markers such as fasting plasma glucose, HDL-C, and triglyceride were analyzed by a biochemical autoanalyzer (Beckman Coulter Synchron system, Lx-20, Fullerton, CA, USA) at the Clinical Laboratory Department of China Medical University Hospital. Fasting plasma glucose was measured in blood obtained by use of NAF TUBE. NAF TUBE contains 5 mg sodium fluoride to inhibit glucose metabolism and 4 mg potassium oxalate to chelate calcium and prevent coagulation. The interassay and intra-assay CVs for fasting plasma glucose were 4% and 4%, respectively. We measured cholesterol and triglyceride in serum mode. A SST tube was used. The SST™ tube refers to the Serum Separator Tube containing clot activator and serum separator gel. The silica particles that coat the walls of the BD Vacutainer^® ^SST™ tube are the clot activator. Initial activation occurs when blood enters the tube and contacts the particles on the tube wall. Triglyceride levels were determined using an enzymatic colorimetric method. The interassay and intra-assay CVs for triglyceride were 6.8% and 5%, respectively. The HDL-C level was measured by a direct HDL-C method and the interassay and intra-assay CVs were 4.5% and 4.5%, respectively. The low-density lipoprotein cholesterol (LDL-C) level was also measured using a direct LDL-C method, and interassay and intra-assay CVs were 4.5% and 3%, respectively. The serum insulin level was measured by a commercial enzyme-linked immunosorbent assay kit (Diagnostic Products, Los Angeles, CA). The interassay CV for insulin was 8.7% and the intra-assay CV was 3.4%. Insulin sensitivity was estimated with a Homeostasis Model Assessment (HOMA-IR) equation. The HOMA-IR equals fasting serum insulin (μU/ml) times fasting plasma glucose (mmol/l) divided by 22.5 [27]. The cut-offs defining abnormality of triglycerides, HDL-C, blood pressure, and fasting glucose were in accordance with the AHA/NHLBI statement that was used to define the four components of MetS [28]: elevated triglycerides (≥150 mg/dL), reduced HDL-C (< 40 mg/dL for men, <50 mg/dL for women), elevated blood pressure (BP ≥130/≥85 mmHg), and elevated fasting glucose (≥100 mg/dL).

Data regarding smoking, alcohol drinking, physical activity, betel nut chewing and family history of cardiovascular-related diseases were collected by questionnaire when the participants underwent a complete physical examination. Subjects who self-reported any of these characteristics (smoking, alcohol drinking, betel nut chewing and family history of cardiovascular-related diseases) were placed into groups based on the specific characteristic. Those in the non-smoking group had never smoked or had smoked less than 100 cigarettes during their lifetime, whereas those in smoking group smoked currently or had smoked more than or equal to 100 cigarettes during their lifetime. Individuals who self-reported alcohol drinking, betel nut chewing or exercise were classified into the group with this specific characteristic. Those whose parents or siblings had a specific cardiovascular-related disease were classified as having a family history of the disease.

### Statistical analysis

Continuous variables were reported as means, whereas categorical variables were reported as numbers (percentage). The cutoff points for WC [28] were 90 (cm) for men and 80 (cm) for women, and for WHR [29] were 0.9 in men and 0.85 in women. The value of the cutoff point of BMI for defining overweight was 24 [30]. We defined the subjects in the upper quartile of the %FM and hs-CRP distributions as the high category group. To examine the relationship between obesity indicators and hs-CRP, crude odds ratios (ORs) were first used; then, multivariate logistic regression analysis was used to investigate the independent effect on the high level of hs-CRP, adjusting for age, smoking, alcohol drinking, and betel nut chewing. Finally, variables for abnormality of the MetS components other than obesity were entered into models. ORs and their 95% confidence intervals were calculated. The areas under curve (AUCs) for each receiver operating characteristics curve were calculated to compare the relative ability of obesity indicators to correctly classify subjects with a high level of hs-CRP, and the nonparametric method was used to test whether the AUCs of these four obesity indicators were different [31]. All analyses were conducted using SAS version 9.1 (SAS Institute Inc, Cary, NC). A two-sided significant level of p < 0.05 was reported.

## Results

A total of 1669 subjects (807 men and 862 women) were analyzed in the final model. The sociodemographic and anthropometric characteristics and biomarkers of the obese participants, as defined by %FM, BMI, WHR, and WC, and stratified by gender, are summarized in Tables 1 and 2. Obese participants, both men and women, identified by these four obesity indicators had similar characteristics.

**Table 1:** Prevalence of cardiovascular risk factors in obese individuals as defined by each obesity indicator, and stratified by gender, n (%)

	Male				Female			
		
	Obesity defined by				Obesity defined by			
		
Variables	%FM (≧29.6)	BMI (≧24)	WHR (≧0.9)	WC (≧90 cm)	%FM (≧40.1)	BMI (≧24)	WHR (≧0.85)	WC (≧80 cm)
Obesity	203 (25.15)	478 (59.23)	340 (42.13)	240 (29.74)	222 (25.75)	366 (42.46)	189 (21.93)	250 (29.00)
Smoking^a^	65 (32.02)	139 (29.08)	118 (34.81)	80 (33.33)	8 (3.62)	10 (2.74)	6 (3.17)	7 (2.80)
Drinking^b^	77 (37.93)	189 (39.54)	128 (37.76)	98 (40.83)	16 (7.24)	27 (7.40)	15 (7.94)	17 (6.80)
Betel nut chewing^b^	14 (6.90)	37 (7.76)	27 (8.01)	17 (7.08)	0 (0.00)	0 (0.00)	0 (0.00)	0 (0.00)
Exercise^b^	135 (66.50)	320 (66.95)	221 (65.19)	164 (68.33)	135 (60.81)	236 (64.48)	121 (64.02)	155 (62.00)
Family history of diabetes^c^	48 (23.65)	116 (24.27)	72 (21.18)	53 (22.08)	52 (23.42)	98 (26.78)	46 (24.34)	58 (23.20)
Hyperglycemia	111 (54.68)	231 (48.33)	168 (49.41)	131 (54.58)	97 (43.69)	153 (41.80)	86 (45.50)	114 (45.60)
Hypertriglyceridemia	102 (50.25)	183 (38.28)	143 (42.06)	104 (43.33)	64 (28.83)	101 (27.60)	62 (32.80)	74 (29.60)
Hypertension	174 (85.71)	354 (74.06)	264 (77.65)	201 (83.75)	163 (73.42)	244 (66.67)	129 (68.25)	185 (74.00)
Low HDL cholesterol	136 (67.00)	301 (62.97)	212 (62.35)	151 (62.92)	157 (70.72)	248 (67.76)	131 (69.31)	171 (68.40)
High level of hs-CRP	74 (36.45)	137 (28.66)	104 (30.59)	76 (31.67)	105 (47.30)	140 (38.25)	83 (43.92)	109 (43.60)

%FM: percent body fat mass, BMI: body mass index, WHR: waist-to-hip ratio, WC: waist circumference, high-density lipoprotein (HDL), high-sensitivity C-reactive protein (hs-CRP)

a: Those in the non-smoking group had never smoked or had smoked less than 100 cigarettes during their lifetime, whereas those in the smoking group smoked currently or had smoked more than or equal to 100 cigarettes during their lifetime.

b: Individuals who self-reported the characteristics of alcohol drinking, betel chewing or exercise were classified into the group with this specific characteristic.

c: Those whose parents or siblings had diabetes were classified as having a family history of diabetes.

**Table 2:** Distributions of sociodemographic and biochemical characteristics in obese individuals defined by each obesity indicator, and stratified by gender

	Obesity defined by*							
	
	%FM		BMI		WHR		WC	
	
Variables	Mean	SD	Mean	SD	Mean	SD	Mean	SD
Male								
Age (years)	59.6	12.7	57.4	12.0	60.1	12.4	59.5	12.4
Fasting blood glucose (mmol/L)	6.1	1.7	5.9	1.6	6.1	1.9	6.1	1.8
Fasting insulin (pmol/L)	96.6	61.0	75.4	52.1	76.1	51.2	91.9	58.1
HOMA-IR	3.7	2.7	2.8	2.3	2.9	2.4	3.6	2.7
Triglyceride (mmol/L)	1.8	1.0	1.7	1.4	1.8	1.40	1.8	1.5
HDL-cholesterol (mmol/L)	1.0	0.2	1.0	0.2	1.0	0.2	1.0	0.3
LDL-cholesterol (mmol/L)	3.5	0.9	3.3	0.8	3.4	0.9	3.4	0.9
Diastolic blood pressure (mmHg)	86.1	8.8	83.5	10.1	83.9	9.8	84.9	9.1
Systolic blood pressure (mmHg)	144.9	17.8	139.2	19.1	141.2	19.2	142.9	18.1
Hs-CRP (mg/l)^#^	0.18	1.05	0.16	1.03	0.17	1.04	0.17	1.05

Female								
Age (years)	58.8	10.2	57.0	9.7	58.4	11.5	58.7	10.6
Fasting blood glucose (mmol/L)	6.1	1.9	5.9	1.8	6.0	1.8	6.1	2.0
Fasting insulin (pmol/L)	82.4	54.4	74.5	55.6	83.6	65.7	79.2	60.4
HOMA-IR	3.2	2.6	2.9	2.8	3.3	3.4	3.2	3.2
Triglyceride (mmol/L)	1.5	0.9	1.4	0.8	1.5	1.0	1.5	0.9
HDL-cholesterol (mmol/L)	1.2	0.3	1.2	0.3	1.2	0.3	1.2	0.3
LDL-cholesterol (mmol/L)	3.5	0.8	3.5	0.8	3.4	0.8	3.4	0.8
Diastolic blood pressure (mmHg)	80.8	11.6	79.0	11.7	79.1	11.3	80.1	11.3
Systolic blood pressure (mmHg)	143.3	21.9	139.3	21.6	139.7	22.6	142.0	22.0
Hs-CRP (mg/l) ^#^	0.21	1.05	0.18	1.04	0.20	1.05	0.21	1.05

%FM: percent body fat mass, BMI: body mass index, WHR: waist-to-hip ratio, WC: waist circumference, homeostasis model assessment of insulin resistance (HOMA-IR), high-density lipoprotein (HDL), low-density lipoprotein (LDL), high-sensitivity C-reactive protein (hs-CRP)

#: geometric mean was presented.

*The cutoff values for %FM: ≥29.6 in men and ≥40.1 in women; for BMI: ≥24; for WHR: ≥0.9 in men and ≥0.85 in women; for WC: ≥90 cm in men and ≥80 in women.

Table 3 shows the ORs of the high level of hs-CRP for each obesity indicator for men and women. Without considering any covariates, all ORs were significant, except BMI, and ORs were largest for %FM for both men and women (OR: 1.94, 95% CI: 1.37-2.73 in men; OR: 3.59, 95% CI: 2.59-4.98 in women). After adjusting for age, smoking status, alcohol drinking, and betel nut chewing, the effect of %FM still remained significant (OR: 1.89, 95% CI: 1.33-2.68 in men; OR: 3.34, 95% CI: 2.39-4.68 in women). After further adjusting for hyperglycemia, hypertriglyceridemia, hypertension, and low HDL-C, the OR for %FM became attenuated, but remained significant (1.55, 95% CI: 1.07-2.25 in men; OR: 2.89, 95% CI: 2.03-4.11 in women). In women, the crude ORs for the four obesity indicators were all statistically significant. These ORs remained significant after considering age, smoking, alcohol drinking, betel nut chewing, and the four components of MetS. The high categories of BMI, WHR, and WC were associated with a 2.11-fold (95% CI: 1.51-2.95), 2.07-fold (1.45-2.97) and 2.37-fold (1.67-3.35) increased risk of having a high level of hs-CRP, respectively.

**Table 3:** The odds ratios of a high level of hs-CRP for obesity as defined by %FM, BMI, WHR, and WC^a^

	OR (95%)	
	
Univariate model	Men	Women
BMI	1.36 (0.98, 1.88)	2.68 (1.97, 3.66)

WHR	1.47 (1.07, 2.01)	2.73 (1.94, 3.84)

WC	1.47 (1.05, 2.05)	3.04 (2.21, 4.18)

%FM	1.94 (1.37, 2.73)	3.59 (2.59, 4.98)

Multivariate model§		

BMI	1.45 (1.04, 2.02)	2.56 (1.86, 3.50)

WHR	1.26 (0.90, 1.74)	2.50 (1.77, 3.54)

WC	1.42 (1.01, 1.99)	2.81 (2.02, 3.90)

%FM	1.89 (1.33, 2.68)	3.34 (2.39, 4.68)

Multivariate model&		

BMI	1.25 (0.87, 1.77)	2.11 (1.51, 2.95)

WHR	1.06 (0.76, 1.50)	2.07 (1.45, 2.97)

WC	1.19 (0.83, 1.70)	2.37 (1.67, 3.35)

%FM	1.55 (1.07, 2.25)	2.89 (2.03, 4.11)

Multivariate model&f		

BMI	1.12 (0.93, 1.33)	1.65 (1.39, 1.97)

WHR	1.09 (0.90, 1.31)	1.40 (1.19, 1.66)

WC	1.18 (0.98, 1.41)	1.76 (1.48,2.12)

%FM	1.50 (1.25, 1.80)	1.72 (1.42, 2.10)

%FM: percent body fat mass; BMI: body mass index; WHR: waist-to-hip ratio; WC: waist circumference.

a: The cutoff values for %FM: ≥29.6 in men and ≥40.1 in women; for BMI: ≥24; for WHR: ≥0.9 in men and ≥0.85 in women; for WC: ≥90 cm in men and ≥80 in women.

§: Logistic regression adjusted for age, smoking, alcohol drinking and betel nut chewing.

&: Logistic regression adjusted for age, smoking, alcohol drinking, betel nut chewing, hyperglycemia, hypertriglyceridemia, hypertension and low HDL cholesterol.

f: per 1-SD increment.

**Figure 1:**
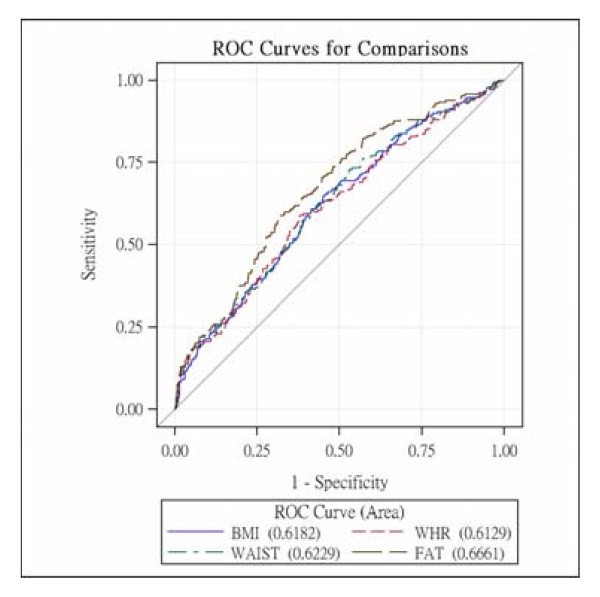
**The receiver operating characteristics curves of BMI, WHR, WC and %FM for males**.

**Figure 2:**
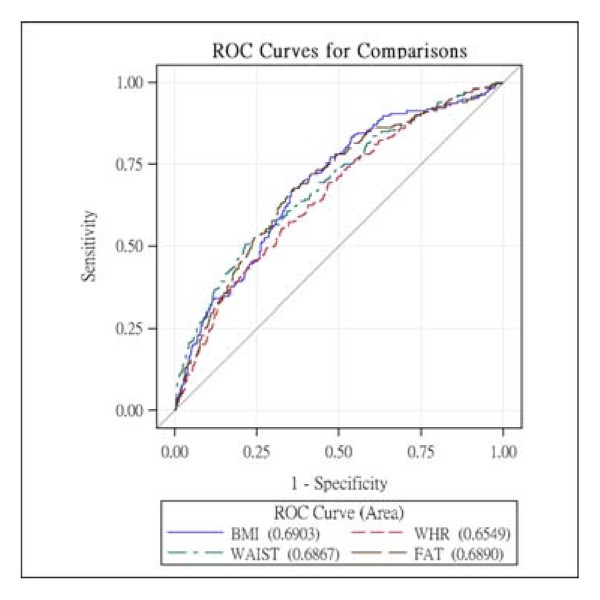
**The receiver operating characteristics curves of BMI, WHR, WC and %FM for females**.

The AUCs and 95% CIs of four obesity indicators stratified by gender are shown in Table 4. In men, the AUCs for BMI, WC, and WHR were similar and were all significantly lower than that for %FM (χ^2 ^for overall test = 14.77, p = 0.002, the p values for pair-wise comparisons were all <0.001, Figure 1). On the other hand, in women, the AUCs for %FM, BMI, and WC were similar and were slightly higher than that for WHR, but there were no significant differences between the AUCs for the different obesity indicators (χ^2 ^= 5.55, p = 0.1355, Figure 2). After further adjusting for the four components of MetS, the differences between the AUCs for the four obesity indicators were no longer statistically significant in men.

**Table 4:** Comparisons of AUCs for percent body fat mass, body mass index, waist-to-hip ratio, and waist circumference, stratified by gender.

	Male			Female		
	AUC	95% CI	*x^2^*	AUC	95% CI	*x^2^*
ROC curve§			14.77**^a^			5.55
%FM	0.67	0.62, 0.71		0.69	0.65, 0.73	
BMI	0.62	0.57, 0.66		0.69	0.65, 0.73	
WHR	0.61	0.57, 0.66		0.65	0.61, 0.70	
WC	0.62	0.58, 0.67		0.69	0.65, 0.73	
ROC curve&			7.74			6.81
%FM	0.68	0.63, 0.72		0.71	0.67, 0.75	
BMI	0.65	0.60, 0.69		0.71	0.67, 0.75	
WHR	0.64	0.60, 0.69		0.69	0.65, 0.73	
WC	0.65	0.60, 0.69		0.71	0.67, 0.75	

%FM: percent body fat mass; BMI: body mass index; WHR: waist-to-hip ratio; WC: waist circumference, AUC: area under curve.

**: p < 0.01;

a: AUC of ROC curve for %FM was significant larger than those for BMI, WHR, and WC

§: Logistic regression adjusted for age, smoking, alcohol drinking and betel nut chewing

&: Logistic regression adjusted for age, smoking, alcohol drinking, betel nut chewing, hyperglycemia, hypertriglyceridemia, hypertension and low HDL cholesterol.

## Discussion

In this community-based study, we demonstrated that all obesity indicators, except WHR in men, were strongly associated with a high CRP level in Taiwanese men and women when sociodemographic factors and lifestyle behaviors were considered. Only the association for %FM remained significant in women after further considering individual abnormalities in the four components of MetS.

Hs-CRP is an easily measured inflammatory biomarker and is released by the liver under the stimulation of cytokines, including interleukin-6, interleukin-1, and tumor necrosis factor-alpha. It has been shown hs-CRP has associations with endothelial dysfunction and insulin resistance syndrome [32]. Although a relationship has been found between hs-CRP and DEXA-measured trunk fat [33], our finding is the first to show that hs-CRP is only associated with %FM in men and women compared with BMI, WHR, and WC, indicating that %FM is the obesity indicator that can capture the inflammatory phenomena in both genders that are responsible for the higher likelihood of diabetes and cardiovascular events. In addition, %FM had a greater ability to classify subjects with a high level of hs-CRP compared with BMI, WHR, and WC in men, when demographic and lifestyle behaviors were considered, but this ability diminished when MetS components were considered. This finding has important implications for obesity screening in community surveys. The BIA measurement may not be more valuable than the other obesity indicators when health surveys use blood samples, but is a measurement worth using when the health survey for screening obesity does not draw blood samples.

WHR has been the traditional anthropometric index for assessing central adiposity; however, the use of WC is gaining support [34-37] and popularity as an alternative, simpler option [38-40]. WHR was the only obesity indicator that did not perform well in men in identifying individuals with a high hs-CRP level after adjusting for age, smoking, alcohol drinking, and betel nut chewing. In Craig's work, WHR did not perform well in identifying risk factors for cardiovascular disease and undiagnosed diabetes [41]. In addition, the correlations with individual risk factors were weaker than those of other measures of body composition, especially in women [41]. On the other hand, WHR explained the highest percentage of the variability of CRP in men in Thorand's work [15], and WHR was significantly correlated with CRP in both men and women [16]. The possible explanation for the differences in the findings among these studies was that these studies were conducted with different ethnic groups, or the populations in these studies had different levels or types of obesity.

The limitations of this study must be considered. The principal limitation relevant to the interpretation of our results is the use of cross-sectional data; thus, causal pathways underlying the observed relationships cannot be inferred. Second, these analyses were restricted to the first 1669 subjects entering the current study, indicating that potential selection bias might exist. To assess this possibility, we examined the demographic characteristics of the individuals with and without hs-CRP measurement by comparing age, sex, and administrative unit, and similar distributions were found. The non-differential distributions in age, sex, and administrative unit indicate this kind of selection error might be random, thus, the biased results in the effect may be toward the null, a lesser threat to validity.

## Conclusion

In summary, this study demonstrates that %FM is strongly associated with a high level of hs-CRP in both genders in a Taiwanese population aged forty years and over. The novelty of the study is that %FM is the only significant indicator related to hs-CRP among various anthrometric indexes in men when demographic and lifestyle behaviors, and the individual components of MetS are considered. This finding highlights the importance of %FM in screening obese individuals who have a higher likelihood of chronic inflammation, especially when no blood sample is drawn. BIA measurement offers a convenient and practical approach to body composition assessment and may be useful and valuable in community settings. Our study finding has important implications for the screening of obesity.

## Abbreviations

hs-CRP: high-sensitivity C-reactive protein; HDL-C: high-density lipoprotein cholesterol; BMI: body mass index; WHR: waist-to-hip ratio; WC: waist circumference; BIA: bio-electrical impedance analysis; %FM: percent body fat mass; DEXA: dual energy X-ray absorptiometry; MetS: metabolic syndrome; ORs: odds ratios; AUCs: receiver operating characteristics curve.

## Competing interests

The authors declare that they have no competing interests.

## Authors' contributions

CCL, SLRK and TCL contributed equally to the design of the study and the direction of its implementation, including supervision of the field activities, quality assurance and control. CIL, CSL, WYL, MML, CCC, TL, CYH, WC and PCC supervised the field activities. CSL and YDL helped conduct the literature review and prepare the Methods and the Discussion sections of the text. TCL, SLRK, CIL, CSL, CCL, CHL and CWY designed the study's analytic strategy and conducted the data analysis. All authors read and approved the final manuscript.

## Pre-publication history

The pre-publication history for this paper can be accessed here:

http://www.biomedcentral.com/1471-2458/10/579/prepub
